# Loss of Atg7 causes chaotic nucleosome assembly of mouse bone marrow CD11b^+^Ly6G^-^ myeloid cells

**DOI:** 10.18632/aging.104176

**Published:** 2020-11-24

**Authors:** Yixuan Fang, Yue Gu, Lei Li, Lingjiang Zhu, Jiawei Qian, Chen Zhao, Li Xu, Wen Wei, Yanhua Du, Na Yuan, Suping Zhang, Ye Yuan, Youjia Xu, Cizhong Jiang, Jianrong Wang

**Affiliations:** 1 Hematology Center of Cyrus Tang Medical Institute, Soochow University School of Medicine, Suzhou 215123, China; 2 National Clinical Research Center for Hematologic Diseases, Collaborative Innovation Center of Hematology, Jiangsu Institute of Hematology, Institute of Blood and Marrow Transplantation, Department of Hematology, The First Affiliated Hospital of Soochow University, Suzhou 215006, China; 3 Department of Hematopoietic Engineering, Susky Life SciTech (Suzhou) Co. Ltd., Suzhou 215124, China; 4 State Key Laboratory of Radiation Medicine and Radioprotection, Soochow University School of Medicine, Suzhou 215123, China; 5 Key Laboratory of Spine and Spinal Cord Injury Repair and Regeneration of Ministry of Education, Orthopaedic Department of Tongji Hospital, the School of Life Sciences and Technology, Shanghai Key Laboratory of Signaling and Disease Research, Tongji University, Shanghai 200092, China; 6 Department of Orthopaedics, the Second Affiliated Hospital of Soochow University, Osteoporosis Institute of Soochow University, Suzhou 215004, China

**Keywords:** Atg7, nucleosome assembly, histone H3.1, aging

## Abstract

Atg7, a critical component of autophagy machinery, is essential for counteracting hematopoietic aging. However, the non-autophagic role of Atg7 on hematopoietic cells remains fundamentally unclear. In this study, we found that loss of Atg7, but not Atg5, another autophagy-essential gene, in the hematopoietic system reduces CD11b myeloid cellularity including CD11b^+^Ly6G^+^ and CD11b^+^Ly6G^-^ populations in mouse bone marrow. Surprisingly, Atg7 deletion causes abnormally accumulated histone H3.1 to be overwhelmingly trapped in the cytoplasm in the CD11b^+^Ly6G^-^, but not the CD11b^+^Ly6G^+^ compartment. RNA profiling revealed extensively chaotic expression of the genes required in nucleosome assembly. Functional assays further indicated upregulated aging markers in the CD11b^+^Ly6G^-^ population. Therefore, our study suggests that Atg7 is essential for maintaining proper nucleosome assembly and limiting aging in the bone marrow CD11b^+^Ly6G^-^ population.

## INTRODUCTION

The functional basic arrangement of eukaryotic DNA is based on the nucleosome, which is comprised of a fragment of DNA that surrounds eight histone proteins. This forms a histone octamer that is comprised of two copies of H2A, H2B, H3, and H4, all of which are histone proteins. Chromosomes are formed by placing several nucleosomes into chromatin, while histones govern the assembly, disassembly, and reassembly of nucleosomes, making them extremely dynamic arrangements [1–3]. The first step to forming nucleosomes and chromatin during the replication of DNA is depositing histones onto early-forming DNA. This nucleosome formation is an important phase, during which the S-phase cells contribute to the synthesis of DNA. This is because cycling cells must quickly insert the nascent DNA within the nucleosome, while simultaneously downplaying the overexpression of histone proteins. To meet this need, cells have evolved several traits related to the formation of nucleosomes and regulatory frameworks that increase the rate at which histones are generated during both normal and stressed cell cycles [4]. As DNA is replicated, the nucleosome is disassembled before the replication machinery, and is subsequently reassembled. H3-H4 proteins are recruited by the association of the replication components and the histone chaperone chromatin assembly factor-1 (CAF-1), which occurs directly on the nascent fragment of DNA [5]. Similarly, the nucleosome assembly protein 1 (NAP-1) inserts histones within the nucleus, which results in the formation of nucleosomes and chromatin fluidity. These processes regulate the transcription of genes. As such, NAP-1 serves an important role in the formation, preservation, and interactions of nucleosomes and chromatin in eukaryotic DNA [6].

Accumulating evidence links aging to genetic and epigenetic alterations [7–9]. However, nucleosome assembly and aging have rarely been connected to each other. A recent study identified Pak2 as a regulator in the deposition of newly synthesized H3.3 onto chromatin, and depletion of Pak2 in mice attenuates the onset of aging-associated phenotypes and extends life span, thereby bridging aging and incorrect deposition of histone H3.3 via Pak2 [10].

Myeloid cells make up a major part of the innate immune response and CD11b^+^Ly6G^−^ myeloid cells have been reported to mediate mechanical inflammatory pain hypersensitivity [11, 12]. Autophagy has been extensively studied and implicated in many aspects of mediating mammalian stem cell aging [13–16]. However, it is unknown if autophagy is linked to aging in differentiated hematopoietic cells such as CD11b^+^Ly6G^−^ myeloid cells and contributes to regulate the dynamics of nucleosome/chromatin assembly. Based on transcriptional profiling and phenotypic analysis of mouse models with autophagy-essential genes selectively deleted in the hematopoietic system, we propose that Atg7 is required to maintain a proper nucleosome/chromatin assembly that may be associated with aging in the bone marrow CD11b^+^Ly6G^-^ myeloid cells.

## RESULTS

### Atg7 deletion diminishes the cellularity of bone marrow CD11b^+^ myeloid cells

**Figure 1:**
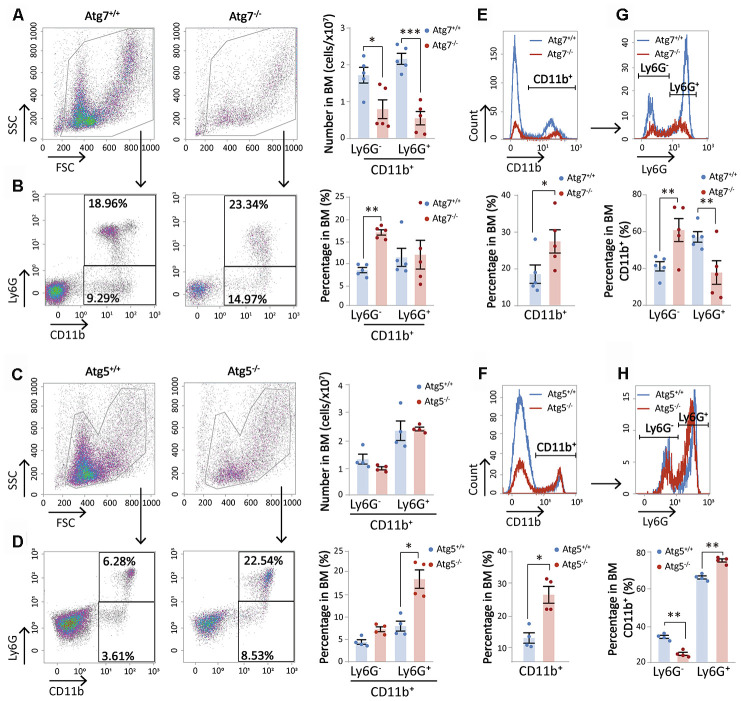
**Atg7 deletion diminishes the cellularity of bone marrow CD11b^+^ myeloid cells.** (**A**–**D**) Graphical and statistical analysis of CD11b and Ly6G by flow cytometry in the bone marrow cells of wild-type and Atg7 or Atg5 hematopoietic-specific deleted mice. (**A**, **C**) Number of CD11b^+^Ly6G^-^ and CD11b^+^Ly6G^+^ myeloid cells in the bone marrow of wild-type and Atg7 or Atg5-deleted mice. (**B**, **D**) Percentage of CD11b^+^Ly6G^-^ and CD11b^+^Ly6G^+^ myeloid cells in the bone marrow of wild-type and Atg7 or Atg5-deleted mice. (**E**, **F**) Percentage of CD11b^+^ and CD11b^-^ myeloid cells in the bone marrow of wild-type and Atg7 or Atg5-deleted mice. (**G**, **H**) Percentage of Ly6G^+^ and Ly6G^-^ myeloid cells in CD11b^+^ myeloid cells from the bone marrow of wild-type and Atg7 or Atg5-deleted mice.

Deletion of Atg7, a key regulator in autophagy, leads to accelerated hematopoietic aging featuring myeloid-biased differentiation [14] and non-hematopoietic organ aging [14, 15]. However, the role of autophagy or autophagy-essential genes on matured hematopoietic cells is not known. To explore specific effects of Atg7 deletion on myeloid cells, we analyzed the pool of CD11b myeloid cells, and their subpopulations sorted with Ly6G. Flow cytometric analysis demonstrated that both total bone marrow CD11b subpopulations of mice with the Atg7 deletion in the hematopoietic system were significantly reduced to around 0.5×10^7^ from approximately 1.7×10^7^ for CD11b^+^Ly6G^-^ and 2.1×10^7^ for CD11b^+^Ly6G^+^. The percentage of CD11b^+^Ly6G^-^ cells over total bone marrow cells increased, apparently due to a higher degree of total bone marrow cell reduction (Figure 1A, 1B).

Deletion of Atg5, another autophagy gene in the hematopoietic system, did not reduce the numbers of either subpopulation in the bone marrow, but increased the percentages of the cells over total bone marrow cells (Figure 1C, 1D). This discrepancy suggests that the reduction of CD11b cellularity caused by Atg7 deletion may not be attributed to the disruption of the Atg7-dependent autophagy. Instead, it suggests an autophagy-independent role of Atg7 in the maintenance of CD11b myeloid cellularity in the bone marrow. Likewise, while the percentage of CD11b cells over total bone marrow cells was significantly increased in both Atg7-deleted mice and Atg5-deleted mice (Figure 1E, 1F), changes in the percentages of CD11b^+^Ly6G^-^ and CD11b^+^Ly6G^+^ over total bone marrow CD11b cells were different between the Atg7 and Atg5-deleted mice. Atg5 deletion caused an opposite change in the percentages of two subpopulations over total CD11b cells as compared to the Atg7-deleted mice (Figure 1G, 1H). This further suggests that Atg7 acts in an autophagy-independent role in maintaining bone marrow CD11b cellularity.

### Atg7-deletion causes abnormal nucleosome assembly of the bone marrow CD11b^+^Ly6G^-^ myeloid cells

**Figure 2:**
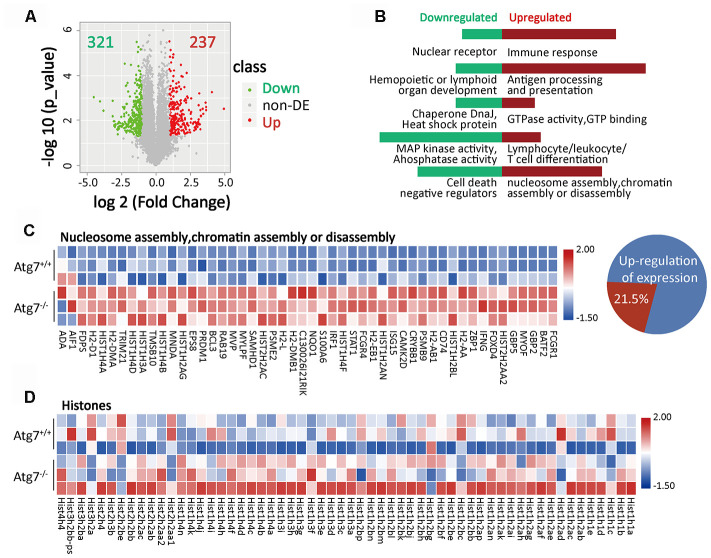
**Atg7-deletion causes abnormal nucleosome assembly of bone marrow CD11b^+^Ly6G^-^ myeloid cells.** (**A**) Volcano plot of differentially expressed genes (fold change >2, *P*-value <0.05) in atg7-deletion compared to wild type CD11b^+^Ly6G^-^ cells. A total of 237 genes were significantly up-regulated, while 321 genes were down-regulated in atg7-deletion CD11b^+^Ly6G^-^ cells. non-DE: non-differentially expressed genes. (**B**) GO enrichment analysis of up-regulated (right) and down-regulated (left) genes. (**C**) Gene expression heatmap of nucleosome/chromatin assembly-associated genes in atg7-deletion and wild type CD11b^+^Ly6G^-^ cells. Percentage of nucleosome/chromatin assembly-associated genes out of total number of up-regulated genes. (**D**) Gene expression heatmap of histone genes in atg7-deletion and wild type CD11b^+^Ly6G^-^ cells.

To determine why CD11b^+^Ly6G^-^ myeloid cells are reduced from Atg7 deletion, we performed RNA sequencing of this cell population in wild-type and Atg7-deleted mice. The volcano plot of differential expression analysis, defined with fold change >2 and *P* value <0.05, shows 321 down-regulated genes and 237 up-regulated genes due to Atg7 deletion (Figure 2A). Gene ontology (GO) enrichment analysis indicated abnormally down-regulated genes involved in nuclear receptors; hematopoietic or lymphoid organ development; chaperone DnaJ; a protein promoting translocation of enzymes [17]; heat shock protein; mitogen-activated protein kinase (MAPK) activity, phosphatase activity; and regulation of cell death (Figure 2B, left). Abnormally upregulated genes were involved in unregulated immune response; antigen processing and presentation; GTPase activity; and lymphocyte/leukocyte/T cell differentiation (Figure 2B, right).

The pathway analysis showed that the capacity of nucleosome assembly or chromatin assembly was upregulated in the Atg7 deleted CD11b^+^Ly6G^-^ cells (Figure 2B, right). Gene expression profiling further indicated that among all 237 abnormally up-regulated genes, 21.5% of the genes are pertinent to nucleosome/chromatin assembly (Figure 2C), suggesting that abnormality of nucleosome/chromatin assembly is the major consequence from loss of the Atg7 gene. Further expression profiling shows that an extensive array of histone members were abnormally upregulated at the transcriptional level in the Atg7 deleted CD11b^+^Ly6G^-^ cells (Figure 2D). These data suggest that Atg7 deletion causes chaotic expression of genes in nucleosome/chromatin assembly.

### Atg7-deletion leads to accumulated histone H3.1 protein with incorrect cytoplasmic localization in the bone marrow CD11b^+^ Ly6G^-^ myeloid cells

**Figure 3:**
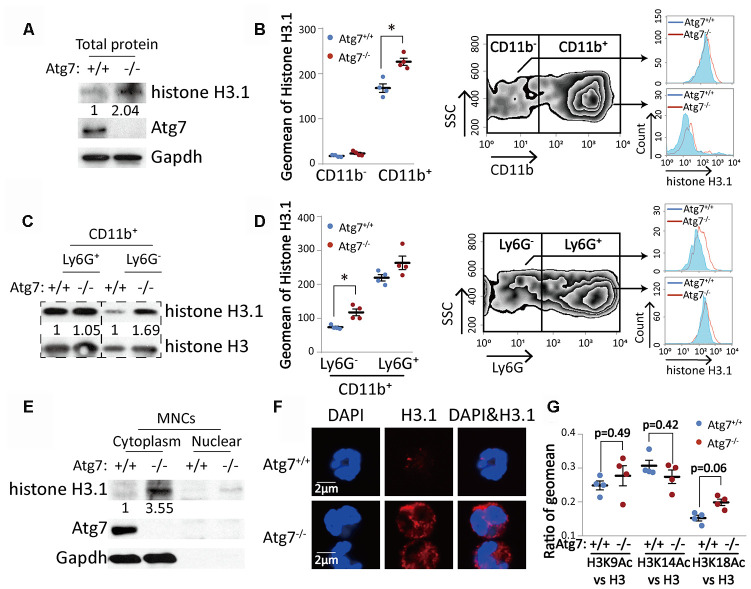
**Atg7-deletion accumulated histone H3.1 protein with incorrect cytoplasmic localization in the bone marrow CD11b^+^Ly6G^-^ myeloid cells.** (**A**, **C**). Western blotting analysis of histone H3.1 in bone marrow cells. Gapdh or total histone H3 was used as a loading control. (**A**) Mononuclear cells; (**C**) CD11b^+^Ly6G^-^ and CD11b^+^Ly6G^+^ myeloid cells. (**B**, **D**) Flow cytometric analysis of protein level of histone H3.1 in bone marrow cells. (**B**) Analysis of histone H3.1 in CD11b^-^ and CD11b^+^ bone marrow cells. (**D**) Analysis of histone H3.1 in CD11b^+^Ly6G^-^ and CD11b^+^Ly6G^+^ myeloid cells. Right, scheme for analysis of histone H3.1 in bone marrow cells. Left, statistical analysis of histone H3.1 geometric mean fluorescence intensity (MFI) in bone marrow cells. (**E**) Western blotting analysis of histone H3.1 in cytoplasm and nucleus from mononuclear cells. (**F**). Confocal detection of histone H3.1 protein in CD11b^+^Ly6G^-^ myeloid cells. (**G**) Ratio of geometric mean of H3K9/14/18Ac compared to H3.

We next examined the protein level of histones, which are the backbone of nucleosomes. Western blotting results showed that Atg7 deletion increased total histone H3.1 in total bone marrow cells (Figure 3A). Flow cytometric analysis further revealed that it was CD11b^+^, not the CD11b^-^ population, increased in protein level (Figure 3B), suggesting abnormal change exclusively in CD11b^+^ myeloid cells. Western blotting results further showed that the increase in histone H3.1 was limited to the CD11b^+^Ly6G^-^ cells (Figure 3C), which was supported by flow cytometric analysis (Figure 3D). Atg7 deletion drove accumulation of H3.1 in the cytoplasm, leaving a minor portion in the nucleus, shown by the Western blotting analysis with bone marrow mononuclear cells (Figure 3E). Furthermore, confocal microscopy showed that Atg7 deletion caused accumulation of H3.1 in the cytoplasm, and this protein was hardly visible in the nucleus (Figure 3F). These results thus suggest that Atg7 deletion resulted in accumulated H3.1 protein trapped in the cytoplasm and caused a failure of H3.1 nuclear localization in the CD11b^+^Ly6G^-^ cell population. To examine if abnormal cytoplasmic localization of H3.1 is caused by changes in acetylation of H3.1, we measured the levels of acetylated H3 proteins. The cytometric results showed that Atg7 deletion did not change acetylation levels of H3 at lysine 9, 14 or 18 (Figure 3G).

### Atg7-deletion results in an aging phenotype in bone marrow CD11b^+^Ly6G^-^ myeloid cells

**Figure 4:**
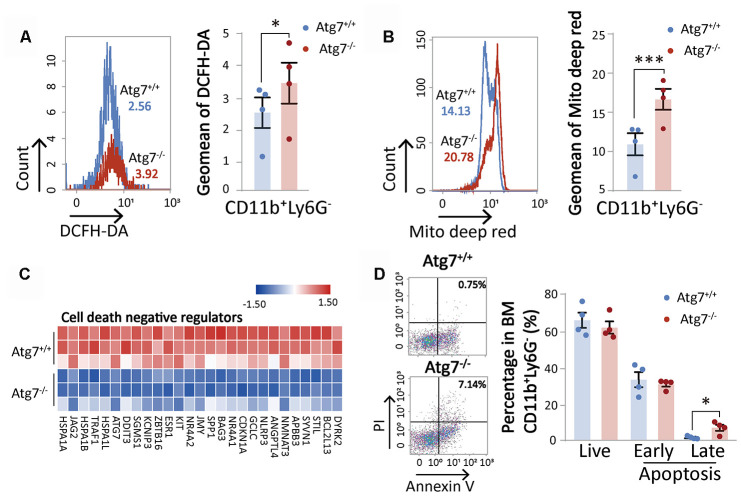
**Atg7 deletion accelerates the aging of CD11b^+^Ly6G^-^ myeloid cells.** (**A**) Flow cytometric analysis of ROS levels of CD11b^+^Ly6G^-^ cells with fluorescent DCFH-DA. Left, histogram for flow cytometric assessment of CD11b^+^Ly6G^-^ cells; right, geometric mean fluorescence intensity (MFI) of DCFH-DA in CD11b^+^Ly6G^-^ cells of wild-type mice and atg7-deleted mice. (**B**) Flow cytometric analysis of mitochondrial mass levels of CD11b^+^Ly6G^-^ cells with florescent MitoTracker Deep Red. Left, histogram for the flow cytometric assessment of CD11b^+^Ly6G^-^ cells; right, geometric MFI of MitoTracker Deep Red in CD11b^+^Ly6G^-^ cells of wild-type and atg7-deleted mice. (**C**) Gene expression heatmap of cell death negative regulators. (**D**) Analysis of apoptosis in CD11b^+^Ly6G^-^ cells of wild-type mice and Atg7-deleted mice by Annexin V and PI double staining. Left, representative flow cytometric measurement; right, statistical results from cytometric analysis. Early apoptosis, Annexin V^+^PI^-^; late apoptosis, Annexin V^+^PI^+^ (right)

Our previous studies established that Atg7-deletion leads to not only speedy aging of the hematopoietic stem and progenitor cells, but also faster aging of non-hematopoietic organs [14, 15]. However, whether differentiated hematopoietic blood cells are affected by loss of Atg7 in terms of aging or lifespan has not been investigated. A functional study with flow cytometry showed that Atg7 deletion increased oxidative stress shown by increased reactive oxygen species (ROS) level (Figure 4A) and mitochondrial mass (Figure 4B) in the myeloid cells. Since mitochondria are a major compartment in the cell that produces ROS, it is likely that the increased ROS is at least partly attributed to the increase of mitochondrial mass. Increased mitochondrial mass and ROS are early triggers that drive cell aging. In the hematopoietic system, senescent cells can be killed by apoptosis [18], suggesting that speedy aging may accelerate programmed cell death. RNA sequencing data showed that Atg7 deletion down-regulated an array of proteins that inhibit the activation of programmed cell death (Figure 4C), and apoptosis was increased from Atg7 deletion (Figure 4D).

## DISCUSSION

**Figure 5:**
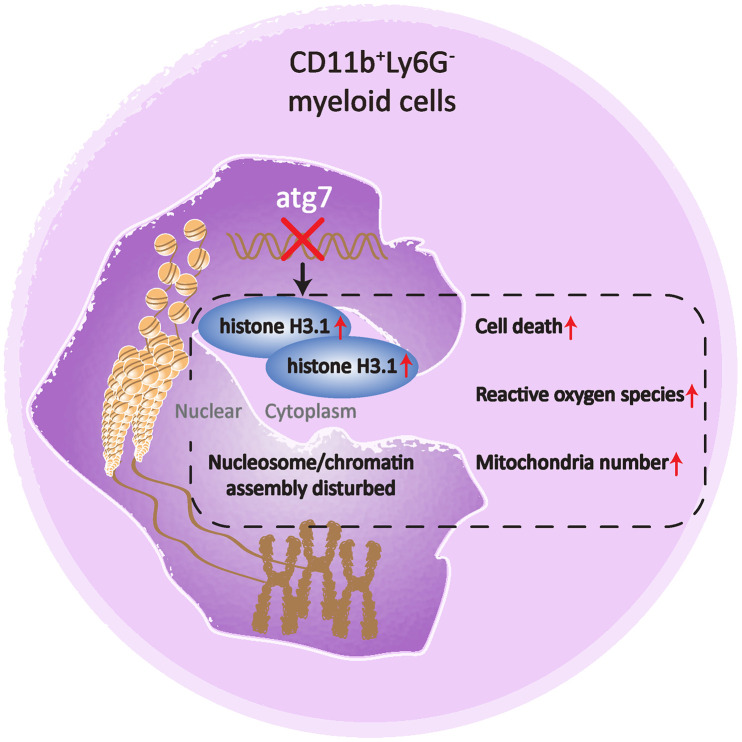
**A cartoon illustrating the role of Atg7 in maintaining proper nucleosome assembly in bone marrow CD11b^+^Ly6G^-^ myeloid cells.** Deletion of Atg7 results in an increased percentage of the CD11b^+^Ly6G^-^ cell population in the bone marrow of mice, accumulation of histone H3.1, cytoplasmic rather than nuclear localization, upregulation of genes related to nucleosome assembly, as well as upregulated aging markers in the CD11b^+^Ly6G^-^ population, thereby suggesting a pivotal role of Atg7 in maintaining proper nucleosome assembly and limiting aging progression in the bone marrow CD11b^+^Ly6G^-^ population.

The present study indicates that Atg7, previously believed to be solely an autophagy-essential gene, is required to maintain proper nucleosome assembly in an autophagy-independent manner in mouse bone marrow CD11b^+^Ly6G^-^ myeloid cells. The abnormality in nucleosome assembly is associated with an increase in the hallmarks for aging and a decrease in cellularity of the Atg7-deleted myeloid cell population (Figure 5).

Recent studies have shown that chromatin organization and remodeling affect aging [7, 19, 20]. Nucleosome positioning regulates chromatin accessibility and is associated with aging [21–23]. RNA sequencing results in this study demonstrated that Atg7 deletion causes chaotic change in the expression of genes involved in nucleosome/chromatin assembly. Among a total of 237 genes that were upregulated in transcription following Atg7 deletion, 21.5% are pertinent to the function of nucleosome assembly (Figure 2), suggesting that dysfunctional nucleosome assembly is a major consequence of Atg7 deletion. Atg7 appears to play a major role in maintaining proper nucleosome assembly in CD11b^+^Ly6G^-^ myeloid cells.

To examine the possible mechanism by which Atg7 supports nucleosome assembly, we measured several major components of nucleosomes. The most significant changes identified were the accumulation of histone H3.1 protein, incorrect localization of the protein in the cytoplasm (Figure 3) and increased oxidative stress (Figure 4). RNA sequencing indicated no change in mRNA level for histone H3.1 in the Atg7-deleted myeloid cells. These findings suggest that loss of Atg7 causes an uncontrolled translation of histone H3.1 or an impaired degradation of histone H3.1 in the cytoplasm. The abnormal accumulation and aberrant localization of histone H3.1 appears not to be caused by epigenetic modification since acetylation levels of H3 were not changed when Atg7 was deleted (Figure 3G).

Aging is driven by excessive oxidative stress [24]. In mitochondria, ROS are tightly regulated by cytochrome c phosphorylation and respirasome factors [25]. Cytochrome c can be shuttled between the mitochondria and the nucleus. Too much cytochrome c impairs DNA remodeling in the nucleus, and thus inhibits nucleosome assembly activity of histone chaperones, such as SET/template-activating factor Iβ and NAP1-related protein during DNA damage in humans [26]. In addition, molecular chaperones have been implicated in the folding of nascent polypeptides, translocation across membranes, and the assembly of oligomeric complexes [27, 28]. Inhibiting the activities of histone chaperones may result in upregulation of genes required in nucleosome assembly to compensate for inhibition of histone chaperones because Atg7 deletion led to enhanced expression of a long array of genes (Figure 2C, 2D), along with enhanced production of oxidative stress, in particular mitochondrial mass (Figure 4A, 4B), which is a major driver for aging progression. Our previous studies also showed that Atg7 deletion increases the membrane potential of the mitochondria [29, 30]. Therefore, more cytochrome c may be released out of the mitochondria and enter the nucleus. That may block DNA remodeling, and ultimately disrupt proper assembly of nuclear DNA and histones into nucleosomes.

Nucleosome assembly proteins (NAPs) directly influence chromatin compaction and modification, including the ability of recruiting nucleosomes to naked DNA templates in chromatin assembly [31, 32]. An earlier study suggested a potential role for MAPK in chromatin reprogramming by histone deacetylase and in chromatin assembly via rapid modification of nucleosome assembly protein 2 (NAP-2/NAP1L4), a homolog of the NAP-1 nucleosome assembly complex subunit [33]. Results showed that MAPK was downregulated in the Atg7-deleted myeloid cells. In order to determine if Atg7 maintains proper nucleosome assembly via MAPK, it would be necessary to examine if nucleosome assembly proteins are influenced by Atg7 deletion in the future.

RNA sequencing data in this study showed that Atg7 deletion leads to down-regulation of heat-shock protein/chaperone DnaJ (Figure 2B). The molecular chaperones of the Hsp70/DnaK family and the cofactors of the DnaJ families play an essential role in protein degradation [34]. DnaJ stimulates the ATPase activity of DnaK [35]. Both components can thus possibly facilitate the recognition of substrate conformations or act as cofactors in the degradative process. It is likely that downregulation of heat-shock protein/chaperone DnaJ may suppress normal degradation of histone H3.1, leading to its accumulation in the cytoplasm.

In summary, our study proposes a role of Atg7 in maintaining nucleosome/chromatin assembly and downregulating aging in myeloid cells. Our future efforts will focus on understanding the mechanism that underlies the link between Atg7 and nucleosome assembly.

## MATERIALS AND METHODS

### Mice

The generation of genetically modified mice Atg7^floxp/floxp^, Atg5^floxp/floxp^ and Vav-iCre have been previously described [30, 36, 37]. Vav-iCre mice were purchased from the Jackson laboratory. Breeding and genotyping of mice were also previously described [14]. Atg7^floxp/floxp^ or Atg5^floxp/floxp^ serves as the control mouse Atg7^+/+^or Atg5^+/+^ in this study. All experimental procedures with animals were approved by Soochow University Institutional Animal Care and Use Committee.

### Flow cytometry

Peripheral blood was collected from the orbit of anesthetized mice. Bone marrow cells are collected from femurs and tibia. Flow cytometry cell sorting and analysis were performed using BD FACSAria^TM^ III and Beckman coulter (gallios). Cell staining procedures were according to the manufacturer’s instruction. The information of all reagents used in this study are given in Table 1.

**Table 1:** Information of the reagents used in this study.

**Name**	**Company**	**Catalog**
CD11b Monoclonal Antibody (M1/70), PE	eBioscience™	12-0112-82
Ly-6G Monoclonal Antibody (1A8-Ly6g), APC	eBioscience™	7-9668-82
Anti-Histone H3.1 antibody	Abcam	ab174712
Recombinant Anti-ATG7 antibody	Abcam	ab133528
GAPDH Antibody	Proteintech	60004-1-Ig
Goat anti-rat IgG (H+L), HRP conjugate	Proteintech	SA00001-15
Goat anti-mouse IgG (H+L), HRP conjugate	Proteintech	SA00001-1
DyLight488 goat anti-mouse IgG [H+L]	Multi Sciences	70-GAM4882
DyLight549 goat anti-mouse IgG [H+L]	Multi Sciences	70-GAM5492
DAPI	Beyotime	C1005
FITC Annexin V Apoptosis Detection Kit	BD Biosciences	556547
Ly-6G Monoclonal Antibody (1A8-Ly6g), PerCP-eFluor 710	eBioscience™	46-9668-82
CM-H2DCFDA (General Oxidative Stress Indicator)	Thermo Fisher Scientific	C6827
MitoTracker™ Deep Red FM	Thermo Fisher Scientific	M22426

### Western blotting

Cells were sorted from mice bone marrow cells and were lysed in 1 X cell lysis buffer (cell signaling technology) with protease inhibitor and phosphatase inhibitor (roche). Protocols of gel electrophoresis, blotting, blocking and treatment with antibodies were previously described [38].

### Immunofluorescence

Cells were sorted by flow cytometry with CD11b positive and Ly6G negative label. CD11b^+^Ly6G^-^ cells were fixed in 4% paraformaldehyde for 15 minutes and permeabilized in 0.5% Triton X-100 for 5 minutes. Then cells were incubated with histone H3.1 antibody overnight at 4° C after blocked with 4% bovine serum albumin for 60 minutes. Cells were treated with secondary antibody and DAPI before photographed on a fluorescence microscope (FV1000MPE-share).

### RNA-Seq

CD11b^+^Ly6G^-^ cells were sorted from 8-week-old Atg7^+/+^ and 8-week-old Atg7^-/-^ mice. Sequencing library were prepared by Novogene. Library preparations were sequencing on an Illumina Hiseq platform and 125 bp/150 bp paired-end reads were generated. HTSeq v0.6.0 [39] was used to count the reads numbers mapped to each gene. Differential expression analysis of two groups was performed using the cuffdiff after removing the batch effect. Genes with an adjusted *P*-value <0.05 and |log2(fold change)|>1 found by DESeq2 were assigned as differentially expressed. Up-regulated or down-regulated genes were processed using biological process GO enrichment. A two-tailed Fisher’s exact test was employed to test the enrichment of the differentially expressed protein against all identified proteins. The GO with a corrected *P*-value < 0.05 is considered significant. In addition, the differentially expressed pattern of genes was analyzed by using R language (v3.4.3). The RNA sequencing data has been deposited in GEO database with an accession number PRJNA634333.

### Statistical analysis

Statistical analyses were performed using SPSS version 22.0. The statistical significance of the observed differences was determined by unpaired t tests. Data were expressed as mean ± standard error of the mean (SEM). *P*<0.05 was considered to indicate a statistically significant difference.
